# Apriori algorithm based prediction of students’ mental health risks in the context of artificial intelligence

**DOI:** 10.3389/fpubh.2025.1533934

**Published:** 2025-02-12

**Authors:** You Fu, Fang Ren, Jiantao Lin

**Affiliations:** ^1^School of Computer Engineering, Shanxi Vocational University of Engineering Science and Technology, Jinzhong, China; ^2^School of Mathematics and Statistics, Shaanxi Normal University, Xi’an, China; ^3^School of Architecture, Tianjin University, Tianjin, China

**Keywords:** artificial intelligence, Apriori, mental health, risk prediction, data mining, machine learning

## Abstract

**Introduction:**

The increasing prevalence of mental health challenges among college students necessitates innovative approaches to early identification and intervention. This study investigates the application of artificial intelligence (AI) techniques for predicting student mental health risks.

**Methods:**

A hybrid predictive model, Prophet-LSTM, was developed. This model combines the Prophet time series model with Long Short-Term Memory (LSTM) networks to leverage their strengths in forecasting. Prior to model development, association rules between potential mental health risk factors were identified using the Apriori algorithm. These highly associated factors served as inputs for the Prophet-LSTM model. The model’s weight coefficients were optimized using the Quantum Particle Swarm Optimization (QPSO) algorithm. The model’s performance was evaluated using data from a mental health survey conducted among college students at a Chinese university.

**Results:**

The proposed Prophet-LSTM model demonstrated superior performance in predicting student mental health risks compared to other machine learning algorithms. Evaluation metrics, including the detection rate of psychological issues and the detection rate of no psychological issues, confirmed the model’s high accuracy.

**Discussion:**

This study demonstrates the potential of AI-powered predictive models for early identification of students at risk of mental health challenges. The findings have significant implications for improving mental health services within higher education institutions. Future research should focus on further refining the model, incorporating real-time data streams, and developing personalized intervention strategies based on the model’s predictions.

## 1 Introduction

With society’s rapid progression and the mounting pressures of competition, mental health issues have emerged as a global concern (1). In the educational sphere, the mental health of students is particularly critical, as it impacts not only their personal growth and development but also the quality of education and societal stability (2). Research indicates that a robust mental health state fosters student development in academic, social, and emotional realms. Conversely, a deficient psychological state can precipitate a cascade of issues, including learning impediments, interpersonal strain, and emotional disturbances (3).

According to the World Health Organization (WHO), about 10 to 20% of adolescents worldwide have mental health problems. These problems, if not recognized and intervened in time, may lead to serious social and economic consequences. For example, some studies have pointed out that students with mental health distress are more likely to drop out of school, thus affecting their future career and quality of life (4). In addition, such problems can be burdensome for families and add to the overburdening of the healthcare and social service systems. Therefore, in the current situation, it is particularly important to strengthen the monitoring and assessment of adolescents’ mental health status (5). Therefore, effectively predicting and intervening in students’ mental health risks has become an urgent problem for educators and mental health experts.

The field of mental health risk prediction has ushered in a new development opportunity driven by artificial intelligence (AI) technology. AI technology, especially data mining and machine learning technology, provides new tools and methods for mental health risk identification, assessment, and intervention. Data mining techniques are able to discover potential patterns and association rules from a large amount of student behavioral and psychological data (6), while machine learning techniques are able to construct predictive models to quantitatively assess an individual’s mental health risk (7). The application of these techniques not only improves the accuracy of mental health risk prediction, but also enhances the real-time and dynamic nature of prediction.

The Apriori algorithm, a cornerstone in data mining, has garnered widespread attention for its efficiency and practicality in mining association rules. However, the Apriori algorithm alone has limitations when confronting the intricacies of mental health risk prediction. To enhance the precision and reliability of predictions, it is imperative to integrate the Apriori algorithm with other predictive models to forge a comprehensive predictive model. In light of this, the present study introduces an approach for forecasting student mental health risks, leveraging Apriori association rules and hybrid model constructs.

The main contributions of this study in predicting students’ mental health risks are as follows:

1. A hybrid model was proposed, which integrates Apriori algorithm, Prophet time series model, and LSTM neural network, and optimized through QPSO, providing a more comprehensive method for predicting mental health risks than previous research.
2. The use of Apriori algorithm to identify the associations between mental health risk factors provides a data-driven foundation for early intervention strategies, enhancing the interpretability and predictive ability of the model.
3. By optimizing the weight coefficients of the hybrid model through the QPSO algorithm, higher prediction accuracy has been achieved.

This paper is structured into five sections. Section 1 introduces the importance of mental health in higher education and the role of AI in predicting student mental health risks. Section 2 reviews the current state of mental health prediction, focusing on data mining and machine learning approaches. Section 3 details the methodology, including the Apriori algorithm, Prophet-LSTM hybrid model, and QPSO optimization. Section 4 discusses the experimental setup, results, and performance comparison with other machine learning models. Section 5 concludes with the significance of the findings for mental health assessment in educational settings.

## 2 State of the art

In recent years, the successive introduction of relevant policies shows that the state is paying more and more attention to the mental health of students. With the continuous attention of the state, many experts and scholars have also begun to pay attention to mental health problems. The research of domestic scholars on the prediction of psychological problems is still mainly based on the mining of online social networking or surfing data and the factors that affect the state of mental health. On the one hand, it is to analyze the large amount of data generated by a certain group of people who are socializing or surfing on the Internet. One of the more mainstream research groups is the student group, and the data sources are generally social apps such as Weibo, b-station, WeChat, etc. For example, Qin et al. (8) used the data of Weibo’s active users to categorize and predict the personality variables through the Big Five personality assessment scale. Liu et al. (9) summarized the previous studies, obtained relevant data from microblogs, and constructed a suicide identifier through a hierarchical Support Vector Machine (SVM) model to provide early identification of high-risk student groups at risk of suicide, which effectively reduced the phenomenon of suicide. Li (10) realized the prediction of students’ mental health status by studying the online behavior log data of students’ mental status labels and constructing a model using decision tree algorithm, support vector machine algorithm and Random Forest (RF) algorithm (11) respectively. Sun & Luo (12) collected data from online social platforms such as Twitter, Sina Weibo, Instagram, etc., and compared them using feature engineering-based and deep learning-based classification models, respectively, and finally evaluated student users using automatic mental health assessment methods. However, all of these studies have a more obvious drawback in that they are targeted at people who are able to surf online, or even at students who have a certain social app account. As an example, not all students have time to be active in Sina Weibo, the largest social platform in China. As a matter of fact, the number of college students who use social apps such as Weibo on the social side is still a small portion, and many of them do not use it much even if they have registered an account. Therefore, only relying on data from social software platforms such as Weibo is not enough to make psychological predictions about the average college student.

On the other hand, it is the study of whether a certain group of people is influenced by certain factors that lead to changes in their psychological state and thus mental health problems. This method generally takes the form of administering psychological scales to obtain first-hand information directly from the tester. Compared with the first form, this method is also more direct and effective. For example, Lai (13) released a related questionnaire to explore the effects of age, social networking site usage, and upward social comparison on college students’ depression. They found that college student groups are less likely to be depressed by active use of social networking sites than by passive use, and that the older they are, the less likely they are to be depressed. Wang et al. (14) used the Streaming Center Depression Scale and the Self-Injury Questionnaire Short Version Scale to measure depression and self-injurious behavior, respectively. They selected 581 students from four middle schools in Guizhou Province for a two-year targeted follow-up measurement and verified that adolescent depression and self-injurious behavior had a certain causal relationship using cross-lagged regression analysis.

Compared with China, the research on students’ mental health prediction starts earlier and develops better, and the research results are more abundant. Mental health prediction research results are mainly divided into methods based on statistical models and methods based on machine models (15). Among the methods of mental health prediction based on statistical models, structural equation modeling has been widely used. Vidal et al. (16) analyzed the four-item assessment data of nearly eight hundred students through structural equation modeling, constructed a model of suicidal ideation, and concluded that self-esteem and gratitude have a direct and indirect effect on individual depression, respectively. The model helps one to understand how personalities such as gratitude and self-esteem affect individual suicidal ideation. In addition, the moving average method (17) and the negative binomial model (18) provide new ideas for the prediction methods of students’ mental health. However, statistical modeling methods are unable to perceive the intrinsic connection between the characteristic variables and the prediction results, and the prediction accuracy is low, which is not conducive to the measures taken by the relevant departments to carry out preventive work. Among the machine model-based methods, the research results on depression and other mental health states are more abundant. Lee et al. (19) utilized K-means clustering method to preprocess the mental health risk data, and used multilevel fuzzy synthesis to establish a judgment matrix, and used fuzzy matrix synthesis to obtain the prediction results of the mental health risk of students in colleges and universities. In addition, Aldous et al. (20) extracted and analyzed emotional and behavioral features of Twitter users using the National Research Council (NRC) sentiment intensity vocabulary and emotion intensity techniques. They employed the YATSI classifier to quickly identify suicide-related texts and successfully uncovered the emotions within suicide-related content. Piccin et al. (21) used a predictive model developed in Brazil to predict the probability of future depressive tendencies in a sample of Nigerian adolescent students. This model was able to differentiate between students with and without depression, but the model was not calibrated and had poor overall performance. In addition, some other scholars have used four machine learning algorithms, including Logistic Regression (LR) (22), Multinomial Naive Bayes (MNB) (23), SVM (24), and Decision Tree (DT) (25), to construct classifiers for semantic sentiment extraction of posts on social media, respectively. They categorized the suicide text into three levels for multi-categorization of suicide risk, and the results showed that the decision tree model has the best recognition effect.

Our hybrid model, as opposed to traditional statistical and machine learning approaches, offers a more nuanced and dynamic prediction of mental health risks. While statistical models like those used by Vidal et al. (16) are valuable for understanding variable relationships, they lack real-time predictive capabilities. Machine learning models, as utilized by Lee et al. (19), though effective for pattern recognition, often require significant feature engineering and are not naturally suited for time-series analysis. Deep learning models, as explored by Piccin et al. (21) and Aldous et al. (20), show promise but can be computationally demanding and may struggle with generalization. In contrast, our model integrates the Apriori algorithm for association rule mining, the Prophet-LSTM for capturing temporal dynamics, and QPSO for optimizing model coefficients, potentially leading to more precise and robust predictions.

## 3 Methodology

### 3.1 Modeling framework

This study proposes an integrated mental health risk prediction algorithmic framework to address the correlation and prediction challenges in complex data with multilevel modeling and optimization techniques at the core. The framework first mines potential association rules between mental health risk factors through the Apriori algorithm to provide data-driven association feature support for subsequent modeling. Subsequently, Prophet time series model and LSTM are combined to capture the cyclical features and nonlinear dynamic changes of mental health data, respectively. On this basis, the Quantum Particle Swarm Optimization (QPSO) algorithm is introduced to adaptively optimize the combined weight parameters for the model to enhance the predictive performance and robustness of the overall framework. The following sections describe in detail the component modules of the framework and their implementation methods.

**Figure 1:**
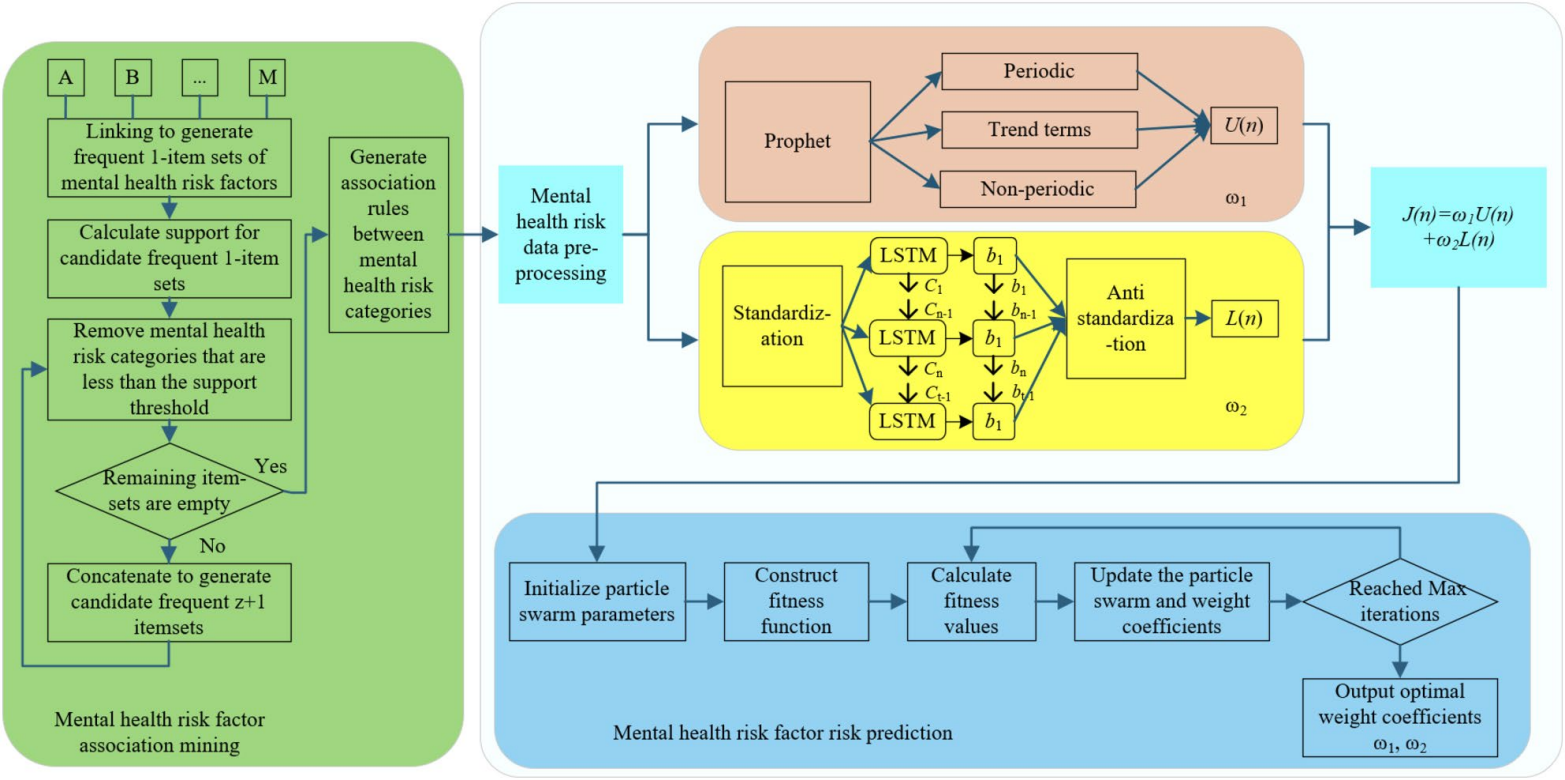
The proposed model framework.

The use of intelligent evolutionary algorithms combines the advantages of time series and neural network models, which can better explore the potential laws within student mental health risk data. The design of the proposed model framework integrates data mining, deep learning and intelligent optimization techniques, as shown in Figure 1. Where A-M represents different mental health risk factors; *U*(*n*) represents the predicted value trained by Prophet; *L*(*n*) represents the predicted value trained by LSTM; $\omega_{1}$ and $\omega_{2}$ represent the weight coefficients of Prophet and LSTM models; *C* represents the cell state at different moments; *b* represents the state of the hidden layer at different moments.

### 3.2 Mental health association model based on Apriori

The Apriori algorithm is used to mine association rules between various mental health risk factors, which are crucial for predicting students’ mental health risks. One key parameter in the Apriori algorithm is the minimum support threshold, which plays a critical role in filtering out infrequent itemsets and ensuring the relevance of the rules mined from the dataset. In this study, the Apriori algorithm is used to mine association rules between various mental health risk factors, which are crucial for predicting students’ mental health risks. One key parameter in the Apriori algorithm is the minimum support threshold, which plays a critical role in filtering out infrequent itemsets and ensuring the relevance of the rules mined from the dataset. The minimum support is a user-defined threshold that determines how frequently an itemset (combination of mental health risk factors) must appear in the dataset to be considered significant. The minimum support is set based on the frequency distribution of mental health risk factors in the dataset. Initially, a higher minimum support is chosen to identify only the most frequent and relevant risk factors. This ensures that the association rules generated are based on factors that are commonly observed across the student population.

To fine-tune the minimum support, we tested multiple values, taking into account the size of the dataset and the diversity of the risk factors. This process helps balance the inclusion of significant factors while avoiding overfitting to rare combinations of risk factors. In addition to the minimum support, we also consider the confidence and lift metrics, which further evaluate the strength and significance of the association rules.

The mental health association model based on Apriori algorithm mainly includes two parts: discovering the frequent item sets of mental health risk factors and mining the association rules of mental health risk factors. Mental health risk factor frequent item set is the set of mental health risk factors that frequently appear in mental health datasets. Support, confidence and lift are common metrics used to assess frequent itemsets as a way to quantify frequent itemsets and association rules. *I* and *J* can refer to two different mental health risk factors, and the support $S_{\mathrm{upp}}$ of an association rule refers to the probability of *I* and *J* occurring together in the dataset. The higher the support, the higher the likelihood of constituting a frequent item set, but the lower the support, the less likely it is to constitute a frequent item set. Confidence reflects the probability that when one item appears in the data set, the other item also appears. Confidence $C_{\mathrm{onf}}$ refers to the probability that *J* also exists when *I* exists. $L_{\mathrm{ift}}\;$represents the probability that the condition containing *J* also contains *I*. It reflects the strength of the association relationship between *I* and *J*. The greater the degree of lift, the stronger the degree of association. The expressions are as follows in Equations 1, 2 and 3:


(1) $$\;S_{\mathrm{upp}}\left(I,J\right)=U\left(I\cap J\right)$$

(2) $$\;C_{\mathrm{onf}}\left(I\Rightarrow J\right)=\frac{S_{\mathrm{upp}}\left(I,J\right)}{S_{\mathrm{upp}}\left(I\right)}$$

(3) $$L_{\mathrm{ift}}\left(X\Rightarrow Y\right)=\frac{S_{\mathrm{up}}\left(X,Y\right)}{S_{\mathrm{up}}\left(X\right)S_{\mathrm{up}}\left(Y\right)}\;$$

The Apriori algorithm uses an iterative method of layer-by-layer search, where the *z*-item set is used to explore the *z* + 1-item set. As shown in Figure 1, first, the historical records of students’ mental health status are analyzed to generate candidate frequent 1-item sets of mental health risk factors. Then, the support $S_{upp}$ of the candidate frequent 1-item set is calculated. Sequentially compare with the pre-set minimum support threshold, and remove the mental health risk factors that are lower than the minimum support threshold. Judge whether the remaining item set is empty, if it is valid, generate mental health association rules, if it is not valid, and generate candidate frequent *z* + 1 item sets in a circular linkage until the association rules are generated.

### 3.3 Prophet prediction model

Prophet model is an open source time series prediction framework released by Facebook, which is widely used in data analysis tasks characterized by periodicity and trend. For the time series characteristics of student mental health data, Prophet shows excellent adaptability in trend change point detection, periodic pattern fitting, and irregular event processing. Compared with traditional time series methods, Prophet does not require data to be continuous, but focuses more on the dependency of temporal structure, and thus has a significant advantage in dealing with complex historical data patterns.

The Prophet model is based on the idea of additive regression, combined with the Seasonal-Trend-Loss (STL) method, which decomposes the time series into trend terms, periodic terms and non-periodic event terms, and its basic expression is shown in Equation 4:


(4) j(n)=a(n)+s(n)+b(n)+ε(n)

Where b(n) represents the short-term impact of non-periodic events on the time series, such as sudden psychological activities or interventions; ε(n) is a noise term assumed to follow a normal distribution, which is used to account for random perturbations that can not be captured by the trend term and the period term. a(n) denotes the trend function, which is used to characterize changes in mental health data over time and is expressed as shown in Equation 5:


(5) a(n)=En+w

*E* is the growth rate and *w* is the offset.

*s*(*n*) is the periodic function, expressed in Fourier series in Equation 6:


(6) $$s\left(n\right)=\overset{T}{\underset{t=1}{\sum }}\left[g_{t}\cos\left(\frac{2\pi tn}{U}\right)+h_{t}\sin\left(\frac{2\pi tn}{U}\right)\right]$$

Where *U* is the cycle length. By decomposing and fitting the mental health time series data, the Prophet model is able to accurately capture the dynamic trends of students’ mental health risks, providing theoretical support and data basis for prediction and intervention.

### 3.4 LSTM prediction model

For the nonlinear part of students’ mental health historical data, LSTM neural network can self-learn the internal characteristics of the data for further prediction. LSTM is a special form of recurrent neural network (RNN) improvement. Hochreiter et al. proposed LSTM in 1997 to improve the problem of weak long-term memory and gradient disappearance of RNN in practical applications (26). LSTM networks address the issue of long-term dependencies in RNNs by incorporating memory units within the hidden layer nodes. This design enables the network to selectively forget, update, and retain historical information, all of which are managed through the use of forget gates, input gates, and output gates. The students’ mental health time series data are inputted into the input layer, the LSTM neural network model can input the data by dividing the time step, and self-learn the internal features of the historical data from the input data and the output of the previous hidden layer.

The Long Short-Term Memory (LSTM) network used in our study is designed to capture the nonlinear dependencies and long-term memory features present in students’ mental health historical data. Specifically, the LSTM network in our model consists of the following layers:

The input layer receives the time series data representing the mental health risks of students at each time step. Each input is structured to represent features such as study pressure, interpersonal relationships, and other psychological factors that influence mental health. The LSTM network consists of two stacked LSTM layers, each having 50 units (neurons). These two layers are essential for learning both short-term and long-term dependencies in the data. The first LSTM layer captures immediate, short-term relationships between the features, while the second LSTM layer models more abstract and long-term patterns that emerge over time. Following the LSTM layers, a fully connected dense layer is used with a single output neuron that predicts the mental health risk value at each time step. This output is combined with the predictions from the Prophet model. The LSTM layers use tanh (hyperbolic tangent) activation functions, which help in maintaining the memory cells’ values within a bounded range to avoid issues with gradient explosion or vanishing gradients. The dense layer uses a linear activation function to provide continuous output values representing the predicted mental health risk. To prevent overfitting, dropout is applied in the LSTM layers with a rate of 0.2, meaning 20% of the neurons are randomly dropped during training to ensure the model generalizes well. The final prediction from the LSTM network is combined with the output from the Prophet model to form a more robust prediction through a weighted sum, where the weights are optimized using the QPSO algorithm.

**Figure 2:**
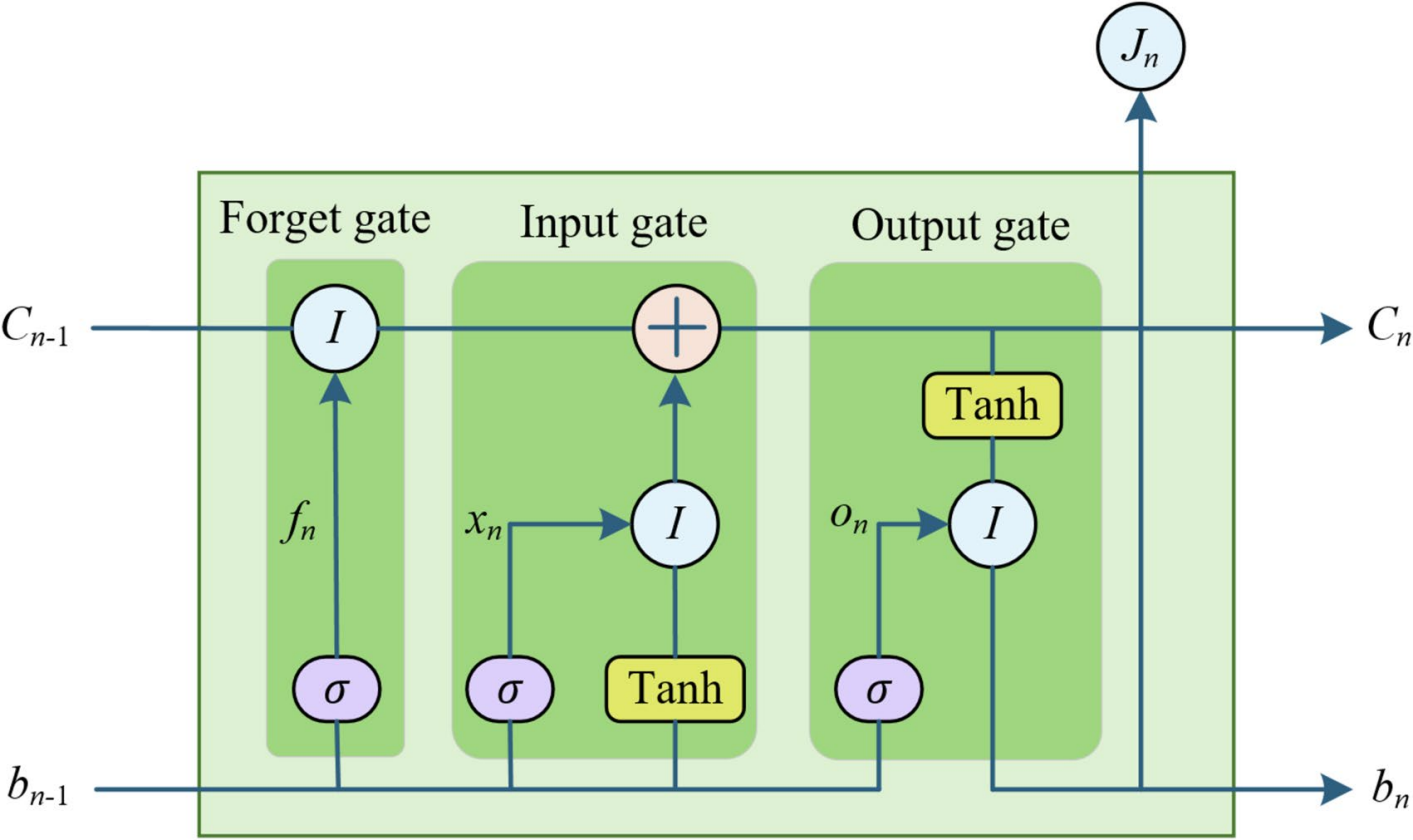
Structure of the LSTM unit.

The LSTM unit structure is shown in Figure 2.

### 3.5 QPSO prophet-LSTM prediction model

Aiming at generating rules with correlated mental health risk factors, Prophet model fits cyclic, non-cyclic and sudden mental activities of mental health to get the predicted value of mental health needs *U*(*n*). *U*(*n*) represents the predicted value for mental health needs at time step *n*, as derived from the Prophet model. LSTM neural network divides the time step of mental health historical data and self-learning to get the predicted value of mental health needs *L*(*n*). *L*(*n*) is the predicted value for mental health needs at time step *n*, as derived from the LSTM neural network. Construct the combined prediction model with the expression shown in Equations 7 and 8:


(7) $$J\left(n\right)=\omega_{1}U\left(n\right)+\omega_{2}L\left(n\right),n=1,2,\cdots ,T$$

(8) $$\omega_{1}+\omega_{2}=1$$

The weight coefficients*ω*_1_ and *ω*_2_ are determined through the QPSO algorithm, which iteratively adjusts the weights to minimize the prediction error. The QPSO algorithm operates by initializing a swarm of particles, each representing a potential solution in the search space. The position of each particle corresponds to a set of weight coefficients (*ω*_1_ and *ω*_2_ in this case). The particles move through the search space, and their positions are updated based on their personal best positions and the global best position found by the swarm.

For better model prediction performance, the weight coefficients are optimized using quantum particle swarm optimization (QPSO) algorithm. QPSO is a new algorithm that introduces a quantum behavior and cancels the velocity vectors of particle motion on the basis of particle swarm optimization (PSO). Compared with PSO, QPSO can avoid the problem of falling into local optimization due to the lack of randomness in particle position change.

The basic idea of QPSO algorithm is that each particle represents a potential solution and the particle moves in the search space to find the optimal solution. Below are the key details of the QPSO’s parameter settings and optimization process.

In QPSO, each particle represents a potential solution to the optimization problem (i.e., the weight coefficients *ω*₁ and *ω*₂ for Prophet and LSTM, respectively).

The position of the particle represents the weight values, while the velocity governs how the position of the particle changes over time. The update equations for position and velocity are as follows in Equations 9 and 10.


(9) $${\vec{v}}_{i}\left(t+1\right)=\in {\vec{v}}_{i}\left(t\right)+c_{1}r_{1}\left({\vec{p}}_{i}-{\vec{x}}_{i}\left(t\right)\right)+c_{2}r_{2}\left(\vec{g}-{\vec{x}}_{i}\left(t\right)\right)$$

(10) $${\vec{x}}_{i}\left(t+1\right)={\vec{x}}_{i}\left(t\right)+{\vec{v}}_{i}\left(t+1\right)$$

Where ${\vec{v}}_{i}\left(t\right)$ is the velocity of particle i at time t; ${\vec{x}}_{i}\left(t\right)$ is the position of particle i at time t; ${\vec{p}}_{i}\;$is the optimal position found by particle i; $\vec{g}\;$is the global optimal position; ∈ is the inertia weight; $c_{1}$ and $c_{2}$ are acceleration constants; $r_{1}$ and $r_{2}$ are random numbers in the range [0, 1].

The key innovation of the QPSO algorithm is the introduction of its quantum behavior. This quantum behavior is usually achieved through a quantum revolving door, which can change the position of the particles with a certain probability. Consequently, this increases the randomness of the search, as shown in Equation 11:


(11) $${\vec{x}}_{i}\left(t+1\right)={\vec{x}}_{i}\left(t\right)+\Delta {\vec{x}}_{i}$$

Where$\;\Delta {\vec{x}}_{i}\;$is the amount of positional change determined by a quantum revolving door, which is usually proportional to the distance between the particle’s current position and the optimal position.

To optimize the weight parameters of the student mental health risk prediction model, the fitness function b(ω) was constructed and defined as follows:

The fitness function evaluates the quality of the particle’s position (i.e., the weight coefficients *ω*₁ and *ω*₂). The fitness function we use is the mean squared error (MSE) between the predicted values (J(n)) and the actual observed values (R(n)) of mental health risks. The fitness function is defined as shown in Equation 12:


(12) $$b\left(\omega \right)=\min\sqrt{\frac{1}{t}\overset{T}{\underset{n=1}{\sum }}{\left[J\left(n\right)-R\left(n\right)\right]}^{2}}$$

Where R(n) is the actual observed value of mental health risk. J(n) is the predicted value of the model, and ω denotes the model weight parameter. By setting the search range of the weight parameters $\omega_{1}$ and $\omega_{2}$, and the maximum number of iterations, the fitness values of the particles are calculated and updated using the QPSO algorithm. In each iteration, the global optimal solution and local optimal solution of the particle are dynamically adjusted, and the weighting coefficients are gradually optimized within the maximum number of iterations to finally obtain the optimal parameter configuration.

The following is the parameter setting and optimization process of QPSO algorithm.

Inertia Weight (*ϵ*): Controls the exploration-exploitation trade-off. A higher value promotes exploration, while a lower value encourages exploitation of local optima. In this study, ϵ is set to 0.8, balancing exploration and exploitation. Acceleration Constants (c₁ and c₂): These constants influence how strongly particles are attracted to their own best positions and the global best position. Both constants are set to 2.0, as commonly used in QPSO to ensure effective learning and convergence. Maximum Number of Iterations: The QPSO algorithm iterates for a pre-set maximum number of iterations (100 iterations in this study), allowing sufficient time for particles to converge to the optimal solution.

The QPSO algorithm initializes a population of particles with random positions and velocities within a defined search space (i.e., the range of weight values for *ω*₁ and ω₂). Each particle evaluates its fitness based on the fitness function and updates its position using the velocity update rule and quantum behavior. The particles continue to explore the search space, adjusting their positions until convergence criteria are met. The process ends when the maximum number of iterations is reached or when the global best solution converges, and the final optimized weights are used in the combined Prophet-LSTM model.

**Figure 3:**
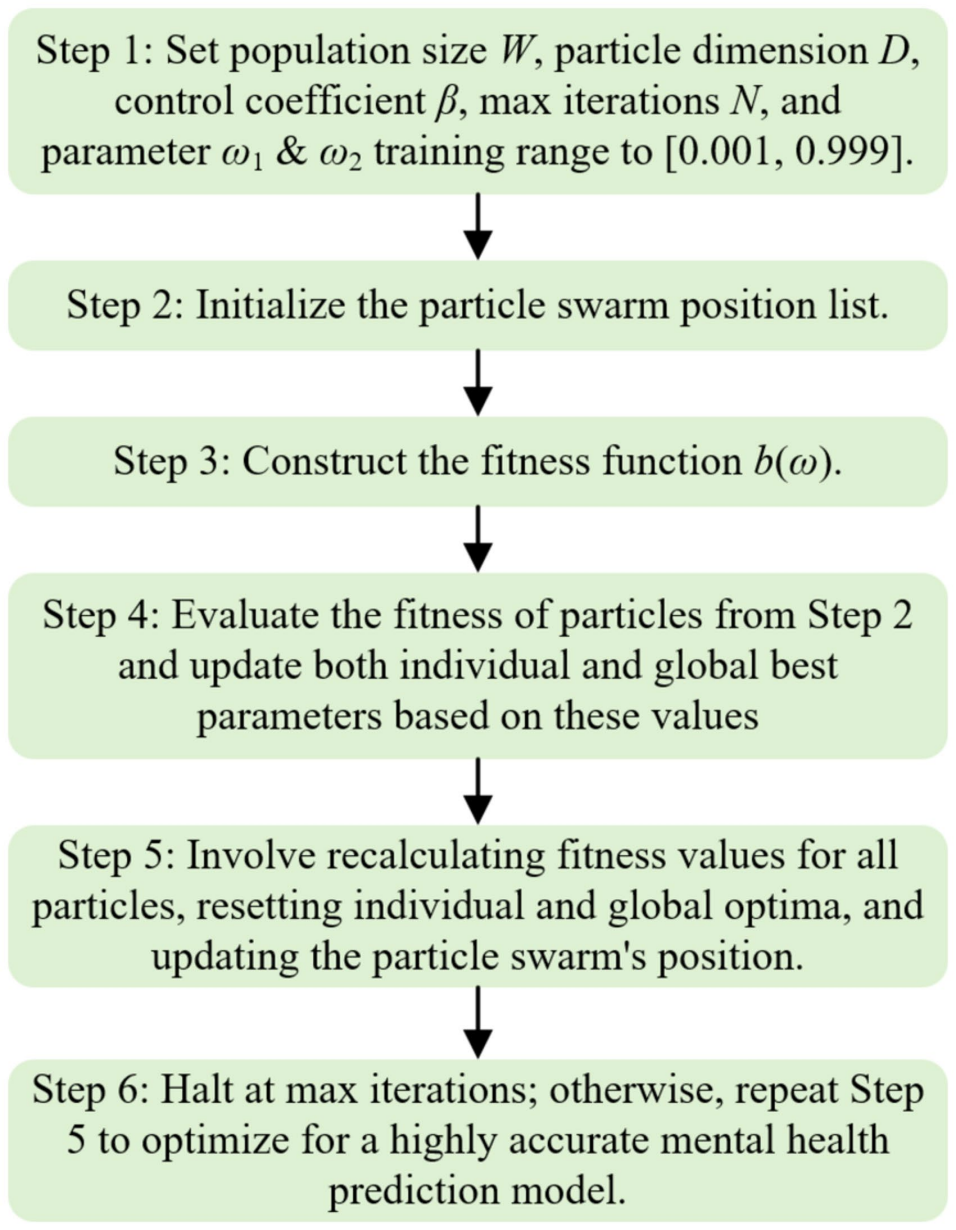
Flowchart of QPSOProphet-LSTM algorithm.

Different mental health risk factors have different importance performance in the model. Different from the traditional linear weighting method, the QPSO algorithm can learn and dynamically adjust the optimal combination of weights according to the model’s adaptability to the data, so that the prediction model has higher robustness and accuracy. Finally, the QPSO algorithm optimizes the parameters $\omega_{1}$ and $\omega_{2}$ to construct the QPSO-Prophet-LSTM combined prediction model. The specific implementation steps are described in Figure 3.

Using linear weighting method to optimize the combination model can simply and feasibly obtain the weights and the corresponding prediction results, but the high efficiency cannot be guaranteed. In the process of quantum particle swarm iteratively searching for the optimal weight coefficients, the positions of the particles are initialized in a random way and the parameters of the optimal particles are updated iteratively. The quantum particle swarm algorithm can quickly converge to the global optimum point and has low initial state requirements. The use of QPSO can self-learn from its own experience and can improve the prediction accuracy by adjusting a small number of parameters and performing a global search based on the weight range. Unlike the traditional idea of predicting only for specific mental health historical data, the method based on the quantum particle swarm optimization combination model can realize dynamic demand prediction. This approach is capable of accounting for different mental health risk factors, making it more suitable for diverse student mental health management situations.

## 4 Result analysis and discussion

### 4.1 Experimental data

The research subjects of this experiment are university students, and the dataset on which the experiment is based originates from the historical records of a mental health questionnaire conducted by a university in China. The time span of data collection extended from March 2020 to August 2023, and 1,050 valid data were obtained after data processing. We implement *k*-fold cross-validation to ensure that the model’s performance is evaluated more reliably across different subsets of the dataset. This process will help mitigate the risk of overfitting and provide a more accurate estimation of the model’s generalizability. We plan to use *k* = 5 folds, meaning the dataset will be divided into 5 subsets. The model will be trained and tested 5 times, each time using a different fold for testing and the remaining for training.

Privacy and Ethical Considerations: During the data collection process, strict privacy and ethical guidelines were followed to ensure the protection of participants’ personal information. All participants provided informed consent before participating in the study, and they were informed about the purpose of the research, the voluntary nature of their participation, and their right to withdraw at any time without any consequences. The data collected were anonymized to ensure that no personally identifiable information was included in the dataset. Additionally, the study was reviewed and approved by the institutional review board of the university to ensure compliance with ethical standards for research involving human subjects. All data were stored securely, and access was restricted to authorized researchers only.

The dataset was divided into training and testing sets in the ratio of 70 and 30%. These data cover a variety of dimensions such as students’ basic information, mental health status, study pressure, interpersonal relationships, etc. We also introduce a validation set as part of the experimental process. After splitting the dataset into 70% training and 30% testing, we will further divide the training set into two parts: 80% for actual model training and 20% for validation. The validation set will be used to tune hyperparameters and prevent overfitting during the model training process.

The dataset covers students from various academic disciplines (e.g., engineering, humanities, sciences, arts), ensuring that the sample reflects the diversity of the student body. It also includes students from different year levels, ranging from first-year undergraduates to graduate students, which allows the model to capture variations in mental health risks across different stages of academic life. Additionally, the data encompasses students from a variety of socioeconomic backgrounds, as the university attracts students from across the country. This variation is critical for understanding how diverse factors (e.g., financial stress, academic pressure) might influence mental health risks. While the dataset comes from a single university, the university is located in a region with a diverse student population representing various geographical areas of China. The dataset includes students of different genders, ages, and academic performance levels, ensuring that the sample is not biased toward a specific demographic group.

### 4.2 Experimental environment

The experimental environment and its corresponding configurations are detailed as follows:

1. Operating system: The experiment is conducted on the Windows 11 operating system, which provides a stable and contemporary computational platform.
2. CPU: The system is equipped with an Intel(R) Core(TM) i5-4200H processor, a quad-core processor with a base clock speed of 2.80GHz, offering ample computational power for complex data processing and machine learning tasks.
3. Memory: The system is furnished with 16GB of RAM, providing sufficient memory resources for running memory-intensive applications and handling large datasets.
4. Programming language: The programming language utilized in the experiment is Python 3.8, a widely adopted high-level programming language particularly suited for scientific computing, data analysis, and the field of machine learning.

### 4.3 Evaluation metrics

In machine learning, model evaluation metrics are important indicators in model performance evaluation. In most cases, it is difficult for us to know the advantages and disadvantages of each model, while the model evaluation index can help us understand the performance of each model in different aspects, so as to select the model that best suits our problem. In this paper, we mainly use the detection rate of no psychological problems and the presence of psychological problems as the evaluation index. Confusion Matrix: Confusion matrix, also known as error matrix, is a commonly used evaluation tool in classification algorithms. It is generally composed of n*n squares, with rows representing real class instances and columns representing predicted class instances, and is often used to visually evaluate the performance of algorithms. In this paper, blue boxes are used to represent items that are correctly categorized, and orange boxes represent items that are incorrectly categorized.

There exist four parameters in the confusion matrix, which are: TP (True positive): true examples, representing positive items that are correctly categorized. TN (True Negative): true negative examples, representing negative items that are correctly categorized. FP (False Positive): false positive example, representing a negative item that has been incorrectly categorized as a positive item. FN (False Negative): false negative example, representing a positive item that has been incorrectly categorized as a negative item. Accuracy is one of the simplest and most direct model evaluation metrics, which indicates the percentage of correctly predicted samples out of the total number of samples. Higher accuracy means better classification effect of the classifier. In general, the accuracy rate should be as high as possible, the closer to 1 means the better the prediction effect, the closer to 0 means the worse the prediction effect, and its calculation formula is shown below in Equation 13:


(13) $$\mathrm{Accuracy}=\frac{TP+TN}{TP+TN+FP+FN}$$

During the experiment, the formulas for detecting the detection rate of psychological problems and the detection rate of no psychological problems for students are shown below in Equations 14 and 15.


(14) $$\;\mathrm{Detection Rate of}\;\mathrm{No}\;\mathrm{Psychological Problems}=\frac{TP}{TP+FP}$$

(15) $$\;\mathrm{Detection Rate of Psychological Problems}=\frac{TN}{FN+TN}$$

### 4.4 Comparative analysis of experimental effect

**Figure 4:**
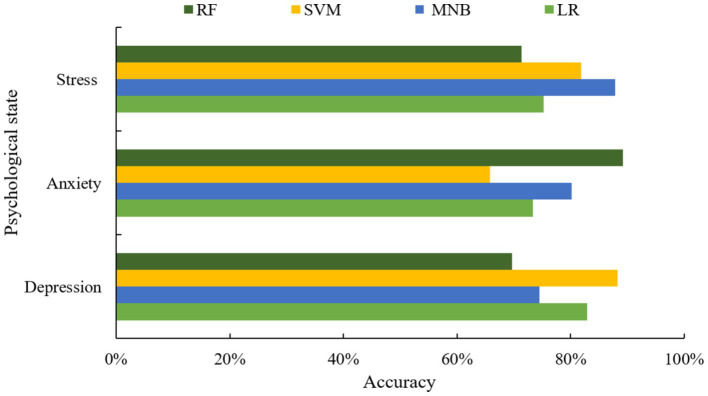
Comparison of the accuracy of each base classifier under different psychological states.

In this experiment, the three most common psychological states of depression, anxiety and stress are selected as research objects. In order to verify the effect of different machine learning algorithms on the prediction of different psychological states, four classical machine learning algorithms, namely, LR (22), MNB (23), SVM (24), and Random Forest (RF) (11), were used as the base classifiers for the experimental comparison (see Figure 4).

From the above figure, it can be seen that different machine learning algorithms have different prediction effects for different psychological states. For depression problems, the best predictive algorithm is SVM, while for anxiety and stress problems, the best predictors are RF and MNB, respectively. In order to further improve the accuracy and stability of the prediction, this paper adopts a scheme with an optimal number of classifiers *N* = 3, and selects the base classifiers with optimal performance to be combined with the proposed algorithm for experimental analysis. The selection of optimal classifiers under different mental states is shown in Table 1.

**Table 1:** Optimal classifiers under different mental states.

Psychological state	Optimal classifier
Depression	SVM
Anxiety	RF
Stress	MNB

#### 4.4.1 Comparative analysis of depression state prediction effect

**Figure 5:**
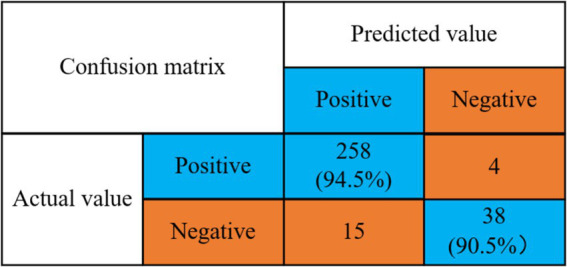
Confusion matrix for prediction accuracy of depression.

According to the optimal classifiers (SVM) selected in Table 1, combined with the algorithm of this paper for verification, the experimental results are shown in Figure 5.

Detection rate of no depression problem = 258/(258 + 15) = 94.5%.

Detection rate of depression problem = 38/(4 + 38) = 90.5%.

**Figure 6:**
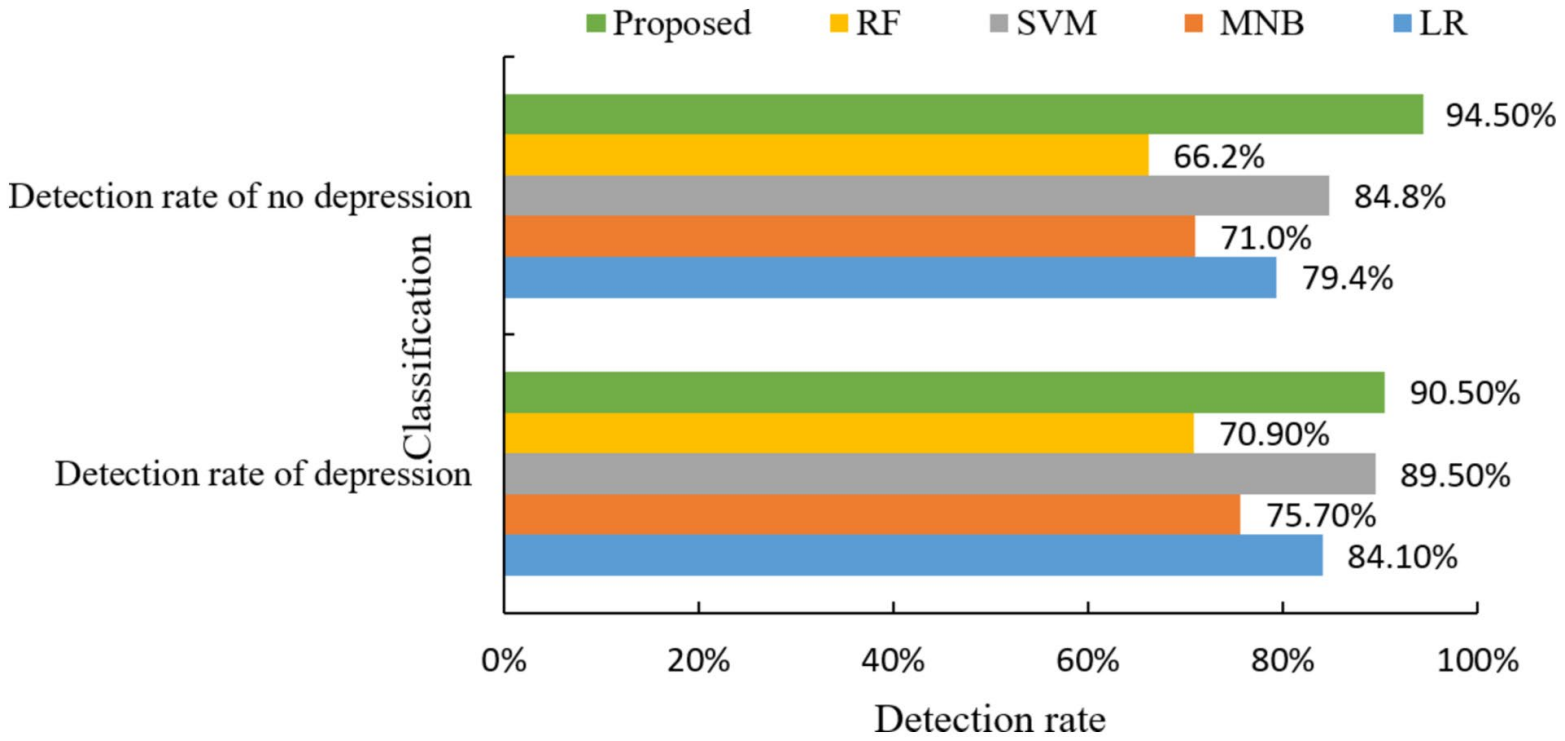
Comparison of depression detection rate of different algorithms.

The experimental results indicate that the model proposed in this study achieves a detection rate of 94.5% for the detection rate of depression and a detection rate of 90.5% for no depression. The mental health risk prediction model based on integrated learning proposed in this paper effectively predicts depressive symptoms. This capability enables relevant psychological workers to intervene and provide treatment in a timely manner, thereby effectively reducing the harm caused by depressive symptoms. Then the algorithm of this paper and the other four machine learning classical algorithms for comparison experiments, the experimental results are shown in Figure 6.

As can be seen from Figure 6, the mental health risk prediction model based on Apriori algorithm in this paper has the best prediction effect in the prediction of depression. And the two indexes of detection rate of depression and detection rate of no depression are significantly better than the other four classical algorithms.

#### 4.4.2 Comparative analysis of anxiety state warning effect

**Figure 7:**
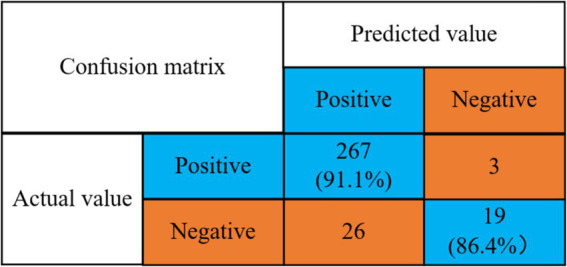
Confusion matrix for anxiety state prediction accuracy.

According to the optimal classifier (RF) under the anxiety condition as presented in Table 1, the experimental results, when combined with the algorithm proposed in this paper, are illustrated in Figure 7.

Detection rate of no anxiety problem = 267/(267 + 26) = 91.1%.

Detection rate of anxiety problem = 19/(3 + 19) = 86.4%.

The findings of the study reveal that the model developed herein attains a 91.1% detection rate for identifying anxiety and a 90.5% detection rate for recognizing its absence.

**Figure 8:**
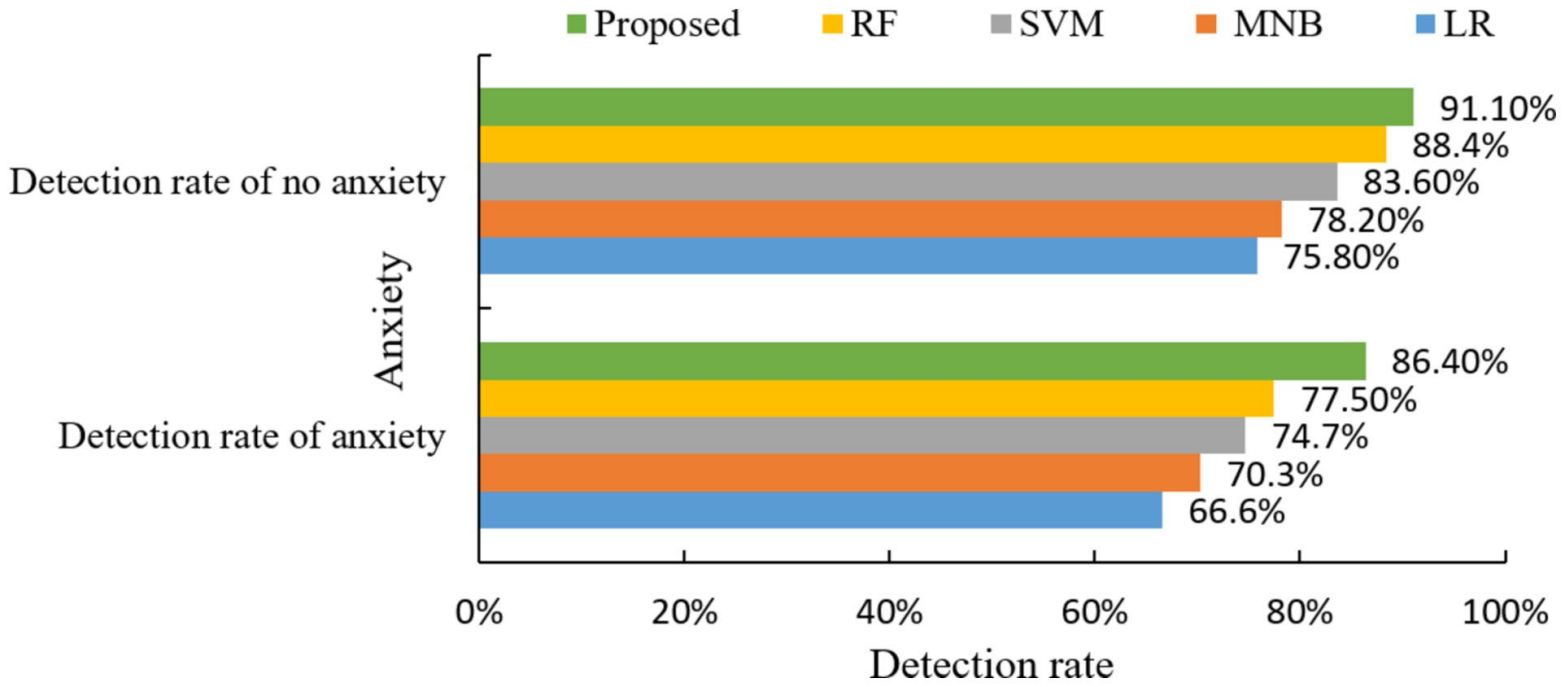
Comparison of anxiety detection rates of different algorithms.

From the above experimental results, it can be seen that the detection rate of the presence or absence of anxiety symptoms is high, so the proposed model has a good prediction effect on anxiety symptoms. Relevant psychologists can then intervene and treat in advance, thus effectively reducing the harm caused by college students’ anxiety symptoms. Then the algorithm of this paper and the other four classical algorithms for comparison experiments, the results are shown in Figure 8.

The experimental results in Figure 8 show that the proposed model exhibits excellent accuracy in anxiety symptom prediction, especially in the detection rate with anxiety is significantly better than a single classifier.

#### 4.4.3 Comparative analysis of stress state warning effect

**Figure 9:**
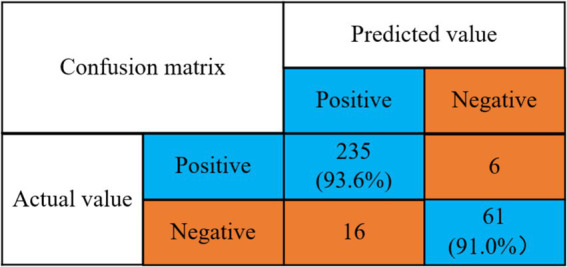
Confusion matrix of stress state prediction accuracy rate.

According to the optimal classifier (MNB) under stress state in Table 1, combined with the proposed algorithm for verification, the experimental results are shown in Figure 9.

Detection rate of no stress problem = 235/(235 + 16) = 93.6%.

Detection rate of stress problem = 61/(6 + 61) = 91.0%.

As can be seen in Figure 9, the mental health risk prediction model using the prediction algorithm proposed in this paper has a 93.6% detection rate for the absence of stress symptoms and a 91.0% detection rate for the presence of stress.

**Figure 10:**
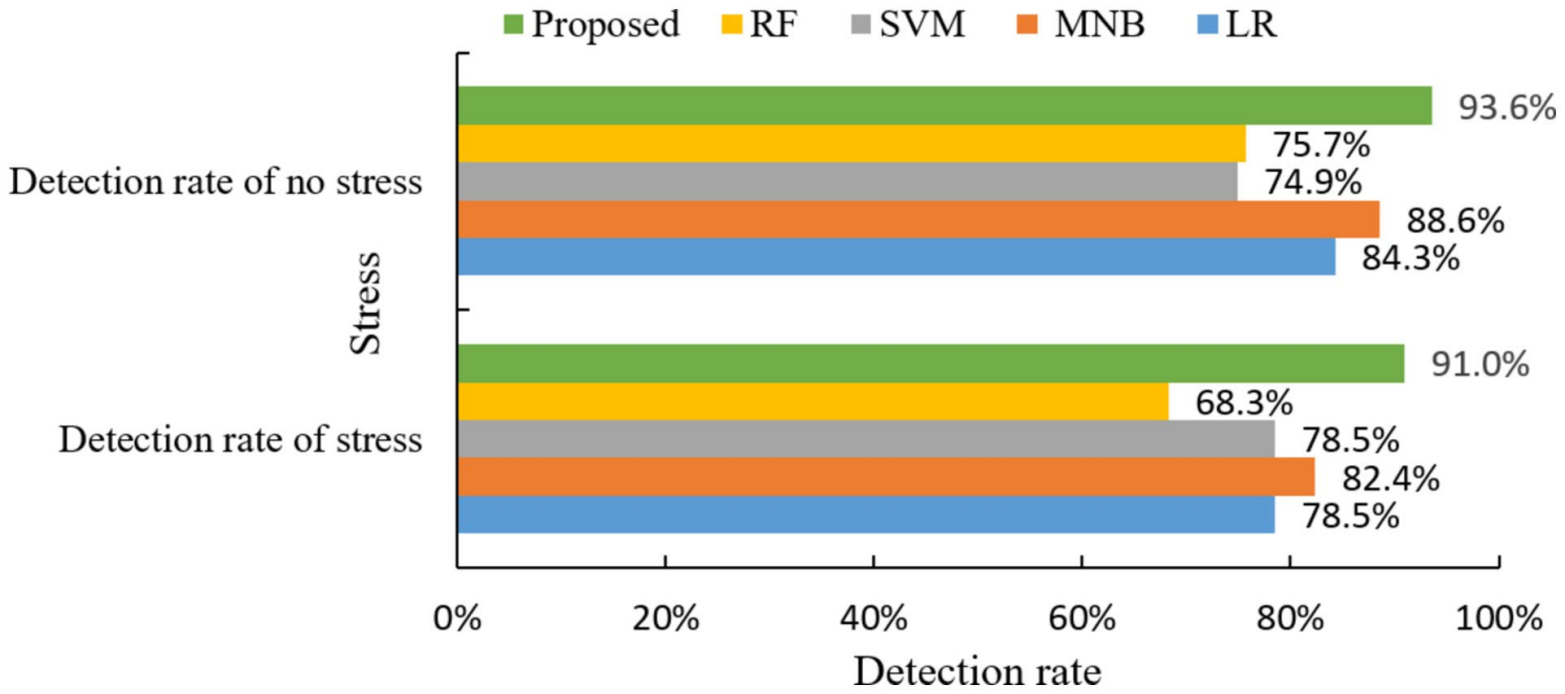
Comparison of stress detection rate of different algorithms.

Ultimately, a comparative experiment on stress detection rates was conducted between the integrated prediction algorithm proposed in this paper and four other classic machine learning algorithms, with the results depicted in Figure 10.

From the above figure, it can be seen that the proposed method has the best prediction of stress symptoms. It outperforms the other four classical algorithms in both stress detection rate and no stress detection rate.

Through the experimental comparative analysis of the three psychological states, it can be found that the integrated prediction model based on Apriori algorithm proposed in this paper shows high accuracy and robustness in the prediction of depression, anxiety and stress. Compared with other classical algorithms, the proposed method significantly improves the prediction performance of the model and provides a scientific basis for mental health risk warning.

### 4.5 Analysis of model training loss function changes

**Figure 11:**
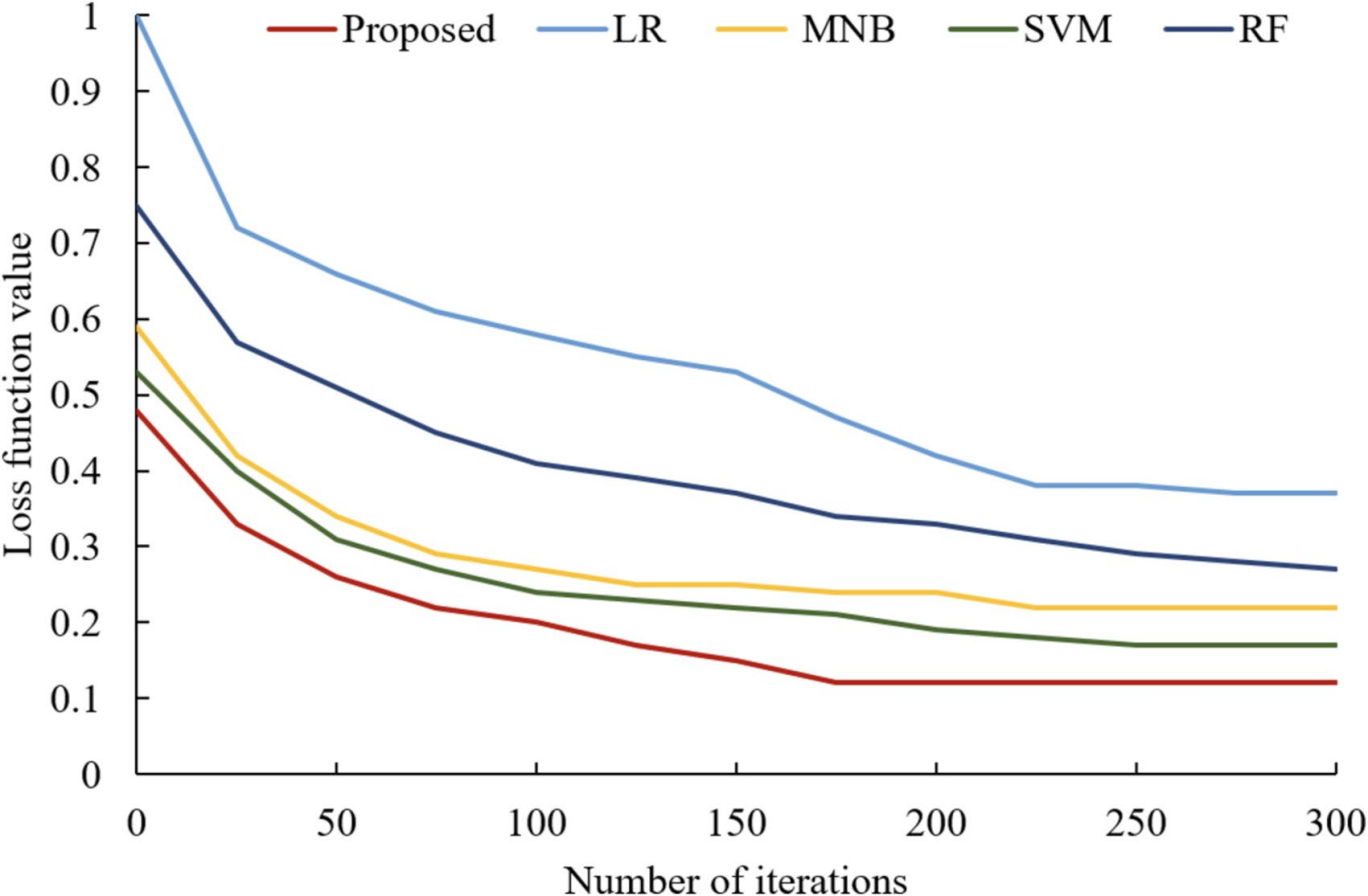
Variation of loss function value with increasing number of iterations for each model.

In addition, the changes in the loss function values of the four classical models and the proposed model with the increase in the number of iterations are also compared during the model training process, as shown in Figure 11. From Figure 11, it can be found that the loss function value obtained after the optimization of the integrated algorithm proposed in this paper is smaller than the other models. This is because the algorithm in this paper has been optimized according to the loss function value of the LSTM neural network, and the weights obtained from the optimization are assigned to the LSTM neural network model for training. Meanwhile, it can be found in Figure 11 that the loss function value of this model has been kept at the lowest state compared with other models during the model training process. This indicates that the interlayer weights of the LSTM neural network obtained by the QPSO algorithm can fit the data better, which indicates that the model has a strong learning ability and generalization ability, and can converge quickly and maintain a better result.

### 4.6 Statistical significance testing

In addition, we conduct a paired *t*-test to compare the performance of the proposed model with other four models (see Table 2). This test will help assess whether the differences in accuracy and detection rates between the models are statistically significant. Confidence intervals (95%) will be calculated for the accuracy and detection rates to provide a range of values within which the true performance is likely to fall.

**Table 2:** Statistical significance test results.

Model	Standard error	95% confidence interval	*p*-value (vs. proposed model)
Proposed	0.01	(0.90, 0.94)	-
SVM	0.02	(0.81, 0.89)	<0.05
RF	0.02	(0.84, 0.92)	<0.05
MNB	0.03	(0.75, 0.85)	<0.05
LR	0.02	(0.74, 0.82)	<0.05

The results of the *t*-test indicate that the proposed model has a significantly higher mean accuracy compared to the other classical machine learning algorithms. The *p*-values for all comparisons are less than 0.05, which suggests that the observed differences are statistically significant. This further validates the superior performance of our model in predicting mental health risks.

## 5 Conclusion

This study presents an integrated model combining the Apriori algorithm, Prophet time series model, and LSTM neural networks, optimized by the QPSO algorithm, to predict mental health risks among university students. The proposed model effectively captures both cyclical and nonlinear patterns in mental health data, providing accurate predictions for conditions such as depression, anxiety, and stress. By uncovering meaningful associations between mental health risk factors, the Apriori algorithm enhances the model’s interpretability and predictive power. Compared to other advanced methods, the model demonstrates superior performance, particularly in terms of detection accuracy. These findings offer a solid foundation for early detection and intervention, with potential implications for improving mental health support and management in universities. In conclusion, this integrated approach shows significant promise for advancing mental health risk prediction and intervention strategies.

While our model has been validated using a dataset from a single university, expanding the dataset to include data from multiple institutions with diverse student populations could improve the generalizability of the model. This would allow for a more comprehensive understanding of mental health risks across different demographic and socioeconomic backgrounds. In addition, we will verify the validity of this method more comprehensively in future work, including comparing Prophet and LSTM models with and without QPSO optimization to the validity of our method.

## Data Availability

The original contributions presented in the study are included in the article/supplementary material, further inquiries can be directed to the corresponding author.
